# 5,6,7,5′-Tetra­meth­oxy-3′,4′-methyl­ene­dioxy­flavone monohydrate

**DOI:** 10.1107/S1600536812015139

**Published:** 2012-04-13

**Authors:** Hou-Jin Li, Da-Lang Zhou, Ting-Juan Xu, Chi-Keung Lam, Wen-Jian Lan

**Affiliations:** a School of Chemistry and Chemical Engineering, Sun Yat-sen University, Guangzhou 510275, People’s Republic of China; b School of Pharmaceutical Sciences, Sun Yat-sen University, Guangzhou 510006, People’s Republic of China

## Abstract

The title compound [systematic name: 5,6,7-trimeth­oxy-2-(7-meth­oxy-1,3-dihydro-2-benzofuran-5-yl)-4*H*-chromen-4-one monohydrate], C_20_H_18_O_8_·H_2_O, was isolated from the popular Chinese medicinal plant *Entada phaseoloides*. In the crystal, inversion-related mol­ecules are joined by pairs of weak C—H⋯O hydrogen bonds. The dimers are further inter­connected by a bridging water mol­ecule *via* weak C—H⋯O_water_ and pairs of (O—H)_water_⋯O hydrogen bonds into a linear tape running parallel to the *b* axis.

## Related literature

For the isolation of 5,6,7,5′-tetra­meth­oxy-3′,4′-methyl­ene­dioxy­flavone, see: Chen *et al.* (1984 ▶); Vyas *et al.* (1986*a* ▶); Souza *et al.* (1995 ▶); Tomazela *et al.* (2000 ▶). For the NMR spectroscopic studies, see Vyas *et al.* (1986*b* ▶). For the biological activity of flavonoids, see: Genoux *et al.* (2011 ▶); Bodewes *et al.* (2011 ▶); Jacob *et al.* (2011 ▶); Veitch & Grayer (2011 ▶). 
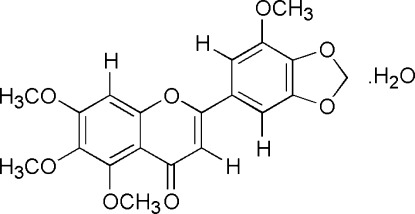


## Experimental

### 

#### Crystal data

C_20_H_18_O_8_·H_2_O
*M* *_r_* = 404.36
Triclinic, 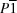

*a* = 9.3014 (17) Å
*b* = 9.3146 (17) Å
*c* = 11.009 (2) Å
α = 105.413 (3)°
β = 91.798 (3)°
γ = 100.985 (3)°
*V* = 899.3 (3) Å^3^
*Z* = 2
Mo *K*α radiation
μ = 0.12 mm^−1^
*T* = 173 K
0.41 × 0.35 × 0.32 mm

#### Data collection

Bruker SMART 1000 CCD diffractometer
Absorption correction: multi-scan (*SADABS*; Bruker, 1998 ▶) *T*_min_ = 0.772, *T*_max_ = 1.000
6560 measured reflections
3181 independent reflections
2608 reflections with *I* > 2σ(*I*)
*R*_int_ = 0.018

#### Refinement

*R*[*F* ^2^ > 2σ(*F* ^2^)] = 0.040
*wR*(*F* ^2^) = 0.117
*S* = 1.07
3181 reflections
270 parameters
4 restraints
H atoms treated by a mixture of independent and constrained refinement
Δρ_max_ = 0.49 e Å^−3^
Δρ_min_ = −0.32 e Å^−3^

### 

Data collection: *SMART* (Bruker, 1998 ▶); cell refinement: *SAINT* (Bruker, 1998 ▶); data reduction: *SAINT*; program(s) used to solve structure: *SHELXS97* (Sheldrick, 2008 ▶); program(s) used to refine structure: *SHELXL97* (Sheldrick, 2008 ▶); molecular graphics: *SHELXTL* (Sheldrick, 2008 ▶); software used to prepare material for publication: *SHELXL97*.

## Supplementary Material

Crystal structure: contains datablock(s) I, global. DOI: 10.1107/S1600536812015139/bg2446sup1.cif

Structure factors: contains datablock(s) I. DOI: 10.1107/S1600536812015139/bg2446Isup2.hkl

Supplementary material file. DOI: 10.1107/S1600536812015139/bg2446Isup3.cml

Additional supplementary materials:  crystallographic information; 3D view; checkCIF report

**Fig. 1.**
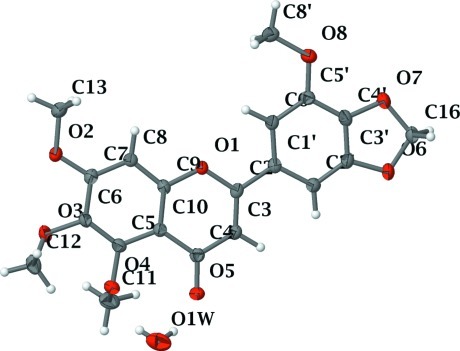
View of the title compound showing displacement ellipsoids at the 50% probability level.

Enhanced figure: interactive version of Fig. 1

## Figures and Tables

**Table 1:** Hydrogen-bond geometry (Å, °)

*D*—H⋯*A*	*D*—H	H⋯*A*	*D*⋯*A*	*D*—H⋯*A*
O1*W*—H11⋯O5	0.83 (2)	2.03 (2)	2.823 (2)	160 (3)
O1*W*—H12⋯O4	0.83 (2)	2.33 (3)	2.987 (2)	137 (3)
C3—H3*A*⋯O5^i^	0.95	2.41	3.255 (2)	147
C8—H8*A*⋯O1*W*^ii^	0.95	2.42	3.372 (2)	175

Symmetry codes: (i) 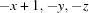; (ii) 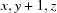.

## supplementary crystallographic information

### Comment

Flavonoids are an important secondary metabolites produced in some medicinal or dietary plants. They are reported to show diverse biological activities, including antioxidant, anti-inflammatory, anti-cancer activities, as well as slowing down the progression of cardiovascular and neurodegenerative diseases (Genoux *et al.*, 2011; Bodewes *et al.*, 2011; Jacob *et al.*, 2011; Veitch *et al.*, 2011).

*Entada phaseoloides* (*L*.) Merr., a popular Chinese medicinal plant, is distributed widely in the South China and the trunk has been used clinically for a long time to treat rheumatism. The title compound, 5,6,7,5'-tetramethoxy-3',4'-methylenedioxyflavone was isolated from the ethanol extract of *Entada phaseoloides*. In the crystals, the methoxy oxygen atom and the adjacent carbonyl oxygen atom are bridged by a water molecule *via* O1w—H11···O5 and O1w—H12···O4 hydrogen bonds. Neighboring molecules related by an inversion centre are joined together *via* a pair of weak C3—H···O5═C4 hydrogen bonds. The molecules are further cross linked by a weak C8'-H···O5═C and C8—H8A···O1w intermolecular interactions.

### Experimental

The trunk of *Entada phaseoloides* (*L*.) Merr. was successively extracted three times with ethanol. The extract was concentrated by low-temperature rotary evaporation and chromatographed on a silica gel column with a petroleum ether–EtOAc–MeOH gradient as the eluent to afford 20 fractions (Fr. 1–Fr. 20). *Fr.* 8–10 were further purified by RP-HPLC eluted with H_2_O–MeCN (60:40, *v*/*v*) to yield 5,6,7,5'-tetramethoxy-3',4'-methylenedioxyflavone. Crystals suitable for X-ray diffraction were obtained by slow evaporation of a solution of the compound in EtOAc.

### Refinement

The H atoms on carbon atoms were placed geometrically and treated as riding on their parent atoms with C—H = 0.98 Å (methyl) or 0.95 Å (aromatic) with *U*_iso_(H) = 1.2*U*_eq_(C). The H atoms of the water molecule were found in a difference Fourier map and refined with an O—H distance restraint of 0.82 (1)Å and free *U*_iso_(H). An antibumping condition between symmetry-related H's was applied in order to preserve a meaningful geometry.

### Crystal data

**Table:**

C_20_H_18_O_8_·H_2_O	*Z* = 2
*M**_r_* = 404.36	*F*(000) = 424
Triclinic, *P*1	*D*_x_ = 1.493 Mg m^−^^3^
Hall symbol: -P 1	Mo *K*α radiation, λ = 0.71073 Å
*a* = 9.3014 (17) Å	Cell parameters from 4354 reflections
*b* = 9.3146 (17) Å	θ = 2.2–27.0°
*c* = 11.009 (2) Å	µ = 0.12 mm^−^^1^
α = 105.413 (3)°	*T* = 173 K
β = 91.798 (3)°	Block, colourless
γ = 100.985 (3)°	0.41 × 0.35 × 0.32 mm
*V* = 899.3 (3) Å^3^	

### Data collection

**Table:**

Bruker SMART 1000 CCD diffractometer	3181 independent reflections
Radiation source: fine-focus sealed tube	2608 reflections with *I* > 2σ(*I*)
Graphite monochromator	*R*_int_ = 0.018
φ and ω scan	θ_max_ = 25.3°, θ_min_ = 2.2°
Absorption correction: multi-scan (*SADABS*; Bruker, 1998)	*h* = −11→11
*T*_min_ = 0.772, *T*_max_ = 1.000	*k* = −11→11
6560 measured reflections	*l* = −13→13

### Refinement

**Table:**

Refinement on *F*^2^	Primary atom site location: structure-invariant direct methods
Least-squares matrix: full	Secondary atom site location: difference Fourier map
*R*[*F*^2^ > 2σ(*F*^2^)] = 0.040	Hydrogen site location: inferred from neighbouring sites
*wR*(*F*^2^) = 0.117	H atoms treated by a mixture of independent and constrained refinement
*S* = 1.07	*w* = 1/[σ^2^(*F*_o_^2^) + (0.0641*P*)^2^ + 0.3125*P*] where *P* = (*F*_o_^2^ + 2*F*_c_^2^)/3
3181 reflections	(Δ/σ)_max_ < 0.001
270 parameters	Δρ_max_ = 0.49 e Å^−^^3^
4 restraints	Δρ_min_ = −0.32 e Å^−^^3^

### Special details

**Table:**

Geometry. All esds (except the esd in the dihedral angle between two l.s. planes) are estimated using the full covariance matrix. The cell esds are taken into account individually in the estimation of esds in distances, angles and torsion angles; correlations between esds in cell parameters are only used when they are defined by crystal symmetry. An approximate (isotropic) treatment of cell esds is used for estimating esds involving l.s. planes.
Refinement. Refinement of *F*^2^ against ALL reflections. The weighted *R*-factor *wR* and goodness of fit *S* are based on *F*^2^, conventional *R*-factors *R* are based on *F*, with *F* set to zero for negative *F*^2^. The threshold expression of *F*^2^ > σ(*F*^2^) is used only for calculating *R*-factors(gt) *etc*. and is not relevant to the choice of reflections for refinement. *R*-factors based on *F*^2^ are statistically about twice as large as those based on *F*, and *R*- factors based on ALL data will be even larger.

### Fractional atomic coordinates and isotropic or equivalent isotropic displacement parameters (Å^2^)

**Table:**

	*x*	*y*	*z*	*U*_iso_*/*U*_eq_	
O1	0.53779 (12)	0.48854 (12)	0.20513 (10)	0.0240 (3)	
O1W	0.4889 (2)	−0.09163 (18)	0.36332 (16)	0.0525 (4)	
H11	0.487 (3)	−0.071 (4)	0.295 (2)	0.093 (11)*	
H12	0.422 (2)	−0.055 (5)	0.3978 (15)	0.23 (3)*	
O2	0.22755 (13)	0.59084 (13)	0.53871 (11)	0.0311 (3)	
O3	0.14023 (13)	0.30245 (14)	0.51890 (12)	0.0341 (3)	
O4	0.25387 (13)	0.08319 (13)	0.34924 (11)	0.0286 (3)	
O5	0.44095 (14)	0.03101 (13)	0.16068 (11)	0.0317 (3)	
O6	0.93016 (15)	0.40373 (14)	−0.22376 (12)	0.0388 (3)	
O7	0.97888 (14)	0.66696 (14)	−0.17464 (12)	0.0363 (3)	
O8	0.85089 (14)	0.85819 (13)	0.01787 (12)	0.0356 (3)	
C2	0.59340 (17)	0.39067 (18)	0.11252 (14)	0.0218 (3)	
C3	0.56048 (18)	0.23941 (18)	0.09729 (15)	0.0241 (4)	
H3A	0.6018	0.1753	0.0316	0.029*	
C4	0.46578 (18)	0.17052 (18)	0.17582 (15)	0.0237 (4)	
C5	0.30091 (18)	0.23401 (18)	0.35496 (15)	0.0234 (4)	
C6	0.24590 (17)	0.34069 (19)	0.44266 (15)	0.0244 (4)	
C7	0.29046 (17)	0.49663 (19)	0.45016 (15)	0.0243 (4)	
C8	0.38842 (17)	0.54320 (18)	0.36968 (15)	0.0229 (4)	
H8A	0.4182	0.6479	0.3738	0.027*	
C9	0.44252 (17)	0.43264 (18)	0.28223 (14)	0.0214 (3)	
C10	0.40336 (17)	0.27721 (18)	0.27200 (15)	0.0215 (3)	
C11	0.1301 (2)	0.0098 (2)	0.2581 (2)	0.0422 (5)	
H11A	0.1008	−0.0971	0.2579	0.063*	
H11B	0.1571	0.0153	0.1739	0.063*	
H11C	0.0479	0.0610	0.2804	0.063*	
C12	0.1733 (2)	0.2124 (2)	0.59780 (18)	0.0384 (5)	
H12A	0.0901	0.1921	0.6476	0.058*	
H12B	0.2611	0.2671	0.6549	0.058*	
H12C	0.1916	0.1158	0.5453	0.058*	
C13	0.2578 (2)	0.7486 (2)	0.54402 (18)	0.0372 (4)	
H13A	0.2063	0.8038	0.6116	0.056*	
H13B	0.2240	0.7610	0.4629	0.056*	
H13C	0.3638	0.7893	0.5612	0.056*	
C16	1.01507 (19)	0.5318 (2)	−0.25493 (16)	0.0295 (4)	
H16A	0.9920	0.5253	−0.3449	0.035*	
H16B	1.1213	0.5340	−0.2413	0.035*	
C1'	0.69092 (17)	0.46871 (18)	0.03700 (15)	0.0222 (3)	
C2'	0.75922 (19)	0.38195 (19)	−0.06075 (16)	0.0270 (4)	
H2'A	0.7408	0.2740	−0.0814	0.032*	
C3'	0.85314 (18)	0.46137 (19)	−0.12415 (15)	0.0261 (4)	
C4'	0.88199 (18)	0.61765 (19)	−0.09635 (15)	0.0257 (4)	
C5'	0.81543 (18)	0.70520 (18)	−0.00182 (15)	0.0247 (4)	
C6'	0.71763 (17)	0.62730 (18)	0.06452 (15)	0.0231 (4)	
H6'A	0.6686	0.6832	0.1294	0.028*	
C8'	0.7778 (2)	0.95003 (19)	0.11035 (17)	0.0326 (4)	
H8'A	0.8132	1.0575	0.1148	0.049*	
H8'B	0.7982	0.9339	0.1931	0.049*	
H8'C	0.6716	0.9218	0.0868	0.049*	

### Atomic displacement parameters (Å^2^)

**Table:**

	*U*^11^	*U*^22^	*U*^33^	*U*^12^	*U*^13^	*U*^23^
O1	0.0278 (6)	0.0202 (6)	0.0242 (6)	0.0049 (5)	0.0109 (5)	0.0057 (5)
O1W	0.0677 (11)	0.0415 (8)	0.0526 (10)	0.0061 (8)	−0.0019 (8)	0.0246 (7)
O2	0.0364 (7)	0.0241 (6)	0.0331 (7)	0.0078 (5)	0.0176 (5)	0.0059 (5)
O3	0.0319 (7)	0.0366 (7)	0.0437 (7)	0.0131 (5)	0.0209 (6)	0.0218 (6)
O4	0.0325 (6)	0.0218 (6)	0.0335 (7)	0.0043 (5)	0.0092 (5)	0.0113 (5)
O5	0.0449 (7)	0.0193 (6)	0.0331 (7)	0.0082 (5)	0.0145 (6)	0.0087 (5)
O6	0.0485 (8)	0.0321 (7)	0.0413 (7)	0.0131 (6)	0.0296 (6)	0.0134 (6)
O7	0.0420 (7)	0.0320 (7)	0.0404 (7)	0.0088 (6)	0.0233 (6)	0.0162 (6)
O8	0.0439 (8)	0.0223 (6)	0.0413 (7)	0.0049 (5)	0.0173 (6)	0.0097 (5)
C2	0.0217 (8)	0.0242 (8)	0.0195 (8)	0.0070 (6)	0.0031 (6)	0.0041 (6)
C3	0.0261 (8)	0.0234 (8)	0.0235 (8)	0.0075 (7)	0.0064 (7)	0.0057 (7)
C4	0.0254 (8)	0.0226 (8)	0.0236 (8)	0.0055 (7)	0.0018 (7)	0.0067 (7)
C5	0.0227 (8)	0.0223 (8)	0.0266 (8)	0.0039 (6)	0.0024 (7)	0.0098 (7)
C6	0.0217 (8)	0.0279 (9)	0.0265 (8)	0.0058 (7)	0.0074 (7)	0.0114 (7)
C7	0.0228 (8)	0.0264 (9)	0.0234 (8)	0.0071 (7)	0.0038 (7)	0.0047 (7)
C8	0.0237 (8)	0.0203 (8)	0.0238 (8)	0.0034 (6)	0.0036 (6)	0.0053 (6)
C9	0.0206 (8)	0.0237 (8)	0.0206 (8)	0.0031 (6)	0.0041 (6)	0.0081 (6)
C10	0.0213 (8)	0.0228 (8)	0.0214 (8)	0.0056 (6)	0.0027 (6)	0.0069 (6)
C11	0.0330 (10)	0.0297 (10)	0.0580 (13)	−0.0048 (8)	0.0017 (9)	0.0104 (9)
C12	0.0452 (11)	0.0461 (11)	0.0305 (9)	0.0121 (9)	0.0147 (8)	0.0186 (9)
C13	0.0461 (11)	0.0255 (9)	0.0400 (10)	0.0096 (8)	0.0178 (9)	0.0057 (8)
C16	0.0284 (9)	0.0347 (10)	0.0293 (9)	0.0097 (7)	0.0120 (7)	0.0125 (7)
C1'	0.0206 (8)	0.0251 (8)	0.0213 (8)	0.0045 (6)	0.0027 (6)	0.0072 (7)
C2'	0.0310 (9)	0.0239 (8)	0.0284 (9)	0.0079 (7)	0.0092 (7)	0.0086 (7)
C3'	0.0273 (9)	0.0299 (9)	0.0239 (8)	0.0108 (7)	0.0083 (7)	0.0083 (7)
C4'	0.0240 (8)	0.0314 (9)	0.0250 (8)	0.0058 (7)	0.0052 (7)	0.0134 (7)
C5'	0.0267 (8)	0.0231 (8)	0.0253 (8)	0.0047 (7)	0.0024 (7)	0.0085 (7)
C6'	0.0232 (8)	0.0258 (8)	0.0213 (8)	0.0076 (7)	0.0042 (6)	0.0063 (7)
C8'	0.0386 (10)	0.0241 (9)	0.0339 (9)	0.0072 (7)	0.0044 (8)	0.0056 (7)

### Geometric parameters (Å, º)

**Table:**

O1—C2	1.3627 (18)	C8—C9	1.393 (2)
O1—C9	1.3723 (19)	C8—H8A	0.9500
O1W—H11	0.829 (17)	C9—C10	1.397 (2)
O1W—H12	0.826 (19)	C11—H11A	0.9800
O2—C7	1.3564 (19)	C11—H11B	0.9800
O2—C13	1.427 (2)	C11—H11C	0.9800
O3—C6	1.369 (2)	C12—H12A	0.9800
O3—C12	1.422 (2)	C12—H12B	0.9800
O4—C5	1.3732 (19)	C12—H12C	0.9800
O4—C11	1.436 (2)	C13—H13A	0.9800
O5—C4	1.240 (2)	C13—H13B	0.9800
O6—C3'	1.374 (2)	C13—H13C	0.9800
O6—C16	1.428 (2)	C16—H16A	0.9900
O7—C4'	1.367 (2)	C16—H16B	0.9900
O7—C16	1.438 (2)	C1'—C6'	1.398 (2)
O8—C5'	1.356 (2)	C1'—C2'	1.411 (2)
O8—C8'	1.426 (2)	C2'—C3'	1.365 (2)
C2—C3	1.347 (2)	C2'—H2'A	0.9500
C2—C1'	1.470 (2)	C3'—C4'	1.376 (2)
C3—C4	1.436 (2)	C4'—C5'	1.383 (2)
C3—H3A	0.9500	C5'—C6'	1.400 (2)
C4—C10	1.465 (2)	C6'—H6'A	0.9500
C5—C6	1.377 (2)	C8'—H8'A	0.9800
C5—C10	1.417 (2)	C8'—H8'B	0.9800
C6—C7	1.412 (2)	C8'—H8'C	0.9800
C7—C8	1.381 (2)		
			
C2—O1—C9	119.79 (12)	O3—C12—H12B	109.5
H11—O1W—H12	103 (3)	H12A—C12—H12B	109.5
C7—O2—C13	117.60 (13)	O3—C12—H12C	109.5
C6—O3—C12	116.78 (13)	H12A—C12—H12C	109.5
C5—O4—C11	112.69 (13)	H12B—C12—H12C	109.5
C3'—O6—C16	106.39 (13)	O2—C13—H13A	109.5
C4'—O7—C16	105.55 (13)	O2—C13—H13B	109.5
C5'—O8—C8'	117.93 (13)	H13A—C13—H13B	109.5
C3—C2—O1	121.29 (14)	O2—C13—H13C	109.5
C3—C2—C1'	125.84 (14)	H13A—C13—H13C	109.5
O1—C2—C1'	112.86 (13)	H13B—C13—H13C	109.5
C2—C3—C4	123.01 (15)	O6—C16—O7	107.82 (13)
C2—C3—H3A	118.5	O6—C16—H16A	110.1
C4—C3—H3A	118.5	O7—C16—H16A	110.1
O5—C4—C3	121.05 (15)	O6—C16—H16B	110.1
O5—C4—C10	124.15 (15)	O7—C16—H16B	110.1
C3—C4—C10	114.80 (14)	H16A—C16—H16B	108.5
O4—C5—C6	118.34 (14)	C6'—C1'—C2'	120.90 (15)
O4—C5—C10	120.27 (14)	C6'—C1'—C2	119.88 (14)
C6—C5—C10	121.39 (14)	C2'—C1'—C2	119.20 (14)
O3—C6—C5	122.89 (14)	C3'—C2'—C1'	116.46 (15)
O3—C6—C7	117.07 (14)	C3'—C2'—H2'A	121.8
C5—C6—C7	119.88 (15)	C1'—C2'—H2'A	121.8
O2—C7—C8	124.83 (15)	C2'—C3'—O6	127.60 (15)
O2—C7—C6	114.65 (14)	C2'—C3'—C4'	123.06 (15)
C8—C7—C6	120.50 (15)	O6—C3'—C4'	109.34 (14)
C7—C8—C9	118.29 (15)	O7—C4'—C3'	110.80 (14)
C7—C8—H8A	120.9	O7—C4'—C5'	127.57 (15)
C9—C8—H8A	120.9	C3'—C4'—C5'	121.61 (15)
O1—C9—C8	114.47 (14)	O8—C5'—C4'	117.04 (15)
O1—C9—C10	122.08 (14)	O8—C5'—C6'	126.13 (15)
C8—C9—C10	123.45 (14)	C4'—C5'—C6'	116.82 (15)
C9—C10—C5	116.48 (14)	C1'—C6'—C5'	121.13 (15)
C9—C10—C4	118.96 (14)	C1'—C6'—H6'A	119.4
C5—C10—C4	124.54 (14)	C5'—C6'—H6'A	119.4
O4—C11—H11A	109.5	O8—C8'—H8'A	109.5
O4—C11—H11B	109.5	O8—C8'—H8'B	109.5
H11A—C11—H11B	109.5	H8'A—C8'—H8'B	109.5
O4—C11—H11C	109.5	O8—C8'—H8'C	109.5
H11A—C11—H11C	109.5	H8'A—C8'—H8'C	109.5
H11B—C11—H11C	109.5	H8'B—C8'—H8'C	109.5
O3—C12—H12A	109.5		
			
C9—O1—C2—C3	−2.1 (2)	C6—C5—C10—C4	−179.51 (14)
C9—O1—C2—C1'	178.78 (12)	O5—C4—C10—C9	178.31 (15)
O1—C2—C3—C4	0.1 (2)	C3—C4—C10—C9	−1.8 (2)
C1'—C2—C3—C4	179.04 (14)	O5—C4—C10—C5	−3.6 (3)
C2—C3—C4—O5	−178.26 (15)	C3—C4—C10—C5	176.28 (14)
C2—C3—C4—C10	1.8 (2)	C3'—O6—C16—O7	−3.22 (18)
C11—O4—C5—C6	93.10 (18)	C4'—O7—C16—O6	2.76 (18)
C11—O4—C5—C10	−86.81 (18)	C3—C2—C1'—C6'	−178.88 (15)
C12—O3—C6—C5	57.9 (2)	O1—C2—C1'—C6'	0.2 (2)
C12—O3—C6—C7	−126.79 (17)	C3—C2—C1'—C2'	−0.4 (2)
O4—C5—C6—O3	−4.0 (2)	O1—C2—C1'—C2'	178.63 (13)
C10—C5—C6—O3	175.91 (14)	C6'—C1'—C2'—C3'	1.0 (2)
O4—C5—C6—C7	−179.23 (14)	C2—C1'—C2'—C3'	−177.43 (14)
C10—C5—C6—C7	0.7 (2)	C1'—C2'—C3'—O6	−178.60 (16)
C13—O2—C7—C8	4.0 (2)	C1'—C2'—C3'—C4'	0.0 (2)
C13—O2—C7—C6	−174.70 (15)	C16—O6—C3'—C2'	−178.74 (17)
O3—C6—C7—O2	3.6 (2)	C16—O6—C3'—C4'	2.46 (18)
C5—C6—C7—O2	179.09 (14)	C16—O7—C4'—C3'	−1.28 (19)
O3—C6—C7—C8	−175.18 (14)	C16—O7—C4'—C5'	179.85 (16)
C5—C6—C7—C8	0.3 (2)	C2'—C3'—C4'—O7	−179.62 (15)
O2—C7—C8—C9	−179.16 (14)	O6—C3'—C4'—O7	−0.7 (2)
C6—C7—C8—C9	−0.5 (2)	C2'—C3'—C4'—C5'	−0.7 (3)
C2—O1—C9—C8	−177.44 (13)	O6—C3'—C4'—C5'	178.20 (15)
C2—O1—C9—C10	2.1 (2)	C8'—O8—C5'—C4'	176.80 (14)
C7—C8—C9—O1	179.29 (13)	C8'—O8—C5'—C6'	−3.2 (2)
C7—C8—C9—C10	−0.3 (2)	O7—C4'—C5'—O8	−1.0 (3)
O1—C9—C10—C5	−178.32 (13)	C3'—C4'—C5'—O8	−179.78 (15)
C8—C9—C10—C5	1.2 (2)	O7—C4'—C5'—C6'	178.95 (15)
O1—C9—C10—C4	−0.1 (2)	C3'—C4'—C5'—C6'	0.2 (2)
C8—C9—C10—C4	179.43 (14)	C2'—C1'—C6'—C5'	−1.5 (2)
O4—C5—C10—C9	178.51 (13)	C2—C1'—C6'—C5'	176.92 (14)
C6—C5—C10—C9	−1.4 (2)	O8—C5'—C6'—C1'	−179.14 (15)
O4—C5—C10—C4	0.4 (2)	C4'—C5'—C6'—C1'	0.9 (2)

### Hydrogen-bond geometry (Å, º)

**Table:**

*D*—H···*A*	*D*—H	H···*A*	*D*···*A*	*D*—H···*A*
O1*W*—H11···O5	0.83 (2)	2.03 (2)	2.823 (2)	160 (3)
O1*W*—H12···O4	0.83 (2)	2.33 (3)	2.987 (2)	137 (3)
C3—H3*A*···O5^i^	0.95	2.41	3.255 (2)	147
C8—H8*A*···O1*W*^ii^	0.95	2.42	3.372 (2)	175

Symmetry codes: (i) −*x*+1, −*y*, −*z*; (ii) *x*, *y*+1, *z*.
